# Experiences From Healthcare and Community Organizations Delivering Evidence-Based Dementia Caregiving Programs

**DOI:** 10.1093/geroni/igab046.027

**Published:** 2021-12-17

**Authors:** Rachel Schaffer, Alyssa Ciancibello, David Bass, Sara Powers

**Affiliations:** Benjamin Rose Institute on Aging, Cleveland, Ohio, United States

## Abstract

Best Practice Caregiving surveyed 324 healthcare and community organizations that replicated one or more of the 44 evidence-based programs about delivery organization characteristics, delivery staff, caregivers and persons with dementia served, funding sources, delivery challenges, perceived impact, and satisfaction. 211 (65.1%) organizations completed surveys about 30 different evidence-based programs. The most common types of organizations that delivered programs were healthcare organizations (23.8%) and Area Agencies on Aging (23.8%). Results showed on average organizations delivered programs for 49 months and served 68 families/year. The most common program delivery challenges were marketing (69.8%) and engaging participants (66.3%). Organizations generally agreed that programs had positive impacts on caregivers (59.5% strongly agree) but were less positive about benefits for persons with dementia (25.1% strongly agree). Discussion provides insights into successes and challenges organizations face when adopting evidence-based dementia caregiving programs in their communities.

